# Scheduled removal of central venous catheters (CVC) to prevent CVC-related bloodstream infections in patients with hematological disease or autologous stem cell transplantation: a registry-based randomized simulation-study

**DOI:** 10.1007/s00277-022-04958-w

**Published:** 2022-08-17

**Authors:** Jens Panse, Daniela Tölle, Eva Fiegle, Jan-Hendrik Naendrup, Martin Schmidt-Hieber, Boris Böll, Marcus Hentrich, Daniel Teschner, Enrico Schalk

**Affiliations:** 1 Department of Oncology, Hematology, Hemostaseology and Stem Cell Transplantation, University Hospital RWTH Aachen, Aachen, Germany; 2 Center for Integrated Oncology (CIO), Aachen, Bonn, Cologne, Düsseldorf (ABCD) Germany; 3 Department of Hematology, Medical Oncology, and Pneumology, University Medical Center of the Johannes Gutenberg University Mainz, Mainz, Germany; 4 Department of Internal Medicine I, University Hospital of Cologne, Cologne, Germany; 5 Clinic of Hematology, Oncology, and Pneumology, Carl-Thiem-Hospital Cottbus, Cottbus, Germany; 6 Department of Hematology and Oncology, Red Cross Hospital Munich, Munich, Germany; 7 Department of Internal Medicine II, University Hospital Würzburg, Würzburg, Germany; 8 Department of Hematology and Oncology, Medical Center, Otto-von-Guericke University Magdeburg, Leipziger Str. 44, 39120 Magdeburg, Germany

**Keywords:** Central venous catheter, Central venous catheter–related bloodstream infection, Prevention, Scheduled removal, Hematology, Registry-based randomized simulation-study

## Abstract

Although not generally recommended, scheduled central venous catheter (CVC) removal is sometimes carried out in order to reduce the CVC-related bloodstream infection (CRBSI) incidence. We conducted a simulation for scheduled CVC removal within the multicenter CRBSI registry (SECRECY). Non-tunneled jugular and subclavian CVC in patients with hematological disease or with germ cell tumors (including patients receiving autologous stem cell transplantation [SCT]) were included. Cases were randomized in a 1:1:1:1 ratio to either a simulated, scheduled CVC removal after 7, 14, and 21 days, or to non-simulated, unscheduled CVC removal (control group). The primary endpoint was definitive CRBSI incidence for a scheduled CVC removal after 14 days (dCRBSI-D14_rmv_). Among other, secondary endpoints were definite CRBSI incidence for a scheduled removal after 7 days (dCRBSI-D7_rmv_) and 21 days ﻿(dCRBSI-D21_rmv_). Data on 2984 CVC were included. Patients’ median age was 59 (range 16–95) years, 58.8% being male. The vast majority (98.4%) were patients with hematological malignancies. Jugular veins were the main insertion site (93.2%). dCRBSI-D14_rmv_ was 3.10/1000 CVC days as compared to 4.15/1000 CVC days in the control group (*p* = 0.23). There was a significant difference between dCRBSI-D7_rmv_ (0.86/1000 CVC days) and controls (*p* < 0.001), but not between dCRBSI-D21_rmv_ (4.10/1000 CVC days) and controls (*p* = 0.96). Our data suggest that in patients with hematological diseases or autologous SCT recipients scheduled CVC removal after 14 days does not result in a lower CRBSI incidence compared to unscheduled removal.

**Trial registration:** DRKS00006551, 2014/09/29, retrospectively registered.

## Introduction

Central venous catheter (CVC)–related bloodstream infections (CRBSI) are potentially preventable complications associated with high morbidity, especially in patients with hematological malignancies [1–3]. The risk for CRBSI is influenced by patient-related, CVC-related, and hospital-related parameters [4, 5]: Patients with hematological malignancies have been shown to be at higher risk for CVC-related infectious complications compared to patients with solid tumors [2], and the risk is associated with the degree and duration of neutropenia [4, 6–8]. In addition, the risk for CRBSI is increased by longer dwelling times of CVC. In a prospective study on 613 neutropenic patients the CRBSI rate was increased by more than 1.5-fold for the entire cohort compared to the rate 14 days after CVC insertion [9]. Thus, prompt removal of CVC no longer required is considered to be an effective measure to reduce CRBSI risk and is recommended in respective guidelines [3, 4].

Although not generally recommended [3, 4, 10], routine CVC replacements are often performed, e.g., every 7 days in intensive care units (ICU) [11, 12]. However, this approach did not reduce the CRBSI incidence [13, 14], because patients with CVC in situ from 1 to 15 days have less risk of bloodstream infection (BSI) than patients with CVC in situ for more than 15 days; e.g. by day 7, 98% of the patients remained free of BSI [15]. A previous study on 1194 CVC cases—mostly patients with hematological malignancies—failed to determine an optimal cutoff time-point at which a prophylactic CVC replacement would prevent CRBSI [16]. In addition, in patients with hematological malignancies, CVCs are often kept in place after administration of intensive chemotherapy regimens for supportive therapies such as intravenous fluids, electrolytes, and transfusions during the vulnerable neutropenic phase despite the fact that they are not absolutely necessary in a strict sense.

The objective of the present study is to add further evidence against a scheduled routine CVC removal in patients with hematological diseases. Given the lack of data from randomized controlled trials (RCT) in the setting of hematological patients [17] a registry-based randomized simulation-study was used.

## Patients and methods

### Registry oversight and study design

The SECRECY registry (Study to Evaluate Central Venous Catheter-related Infections in Hematology and Oncology; German Clinical Trial Register [DRKS], no. DRKS00006551) is an ongoing, clinical CRBSI registry starting in March 2013 including mainly patients with hematological malignancies, along with few patients with solid tumors and benign hematological diseases [18]. Currently, six sites are active in Germany. Surveillance data are collected on CRBSI of all non-selected, consecutive patients with centrally inserted short-term, non-tunneled jugular, subclavian or femoral vein CVC inserted for routine clinical use in adult patients generally treated on hematology and oncology wards. One center, however, also included hematological and oncological patients receiving treatment in an ICU or intermediate care unit. All CVC were inserted according to local standard operating procedures.

CRBSI were classified according to the 2012 Infectious Diseases Working Party (AGIHO) of the German Society of Hematology and Medical Oncology (DGHO) definition [19]. The microbiological specimens were investigated and analyzed at the local sites and local investigators assessed the CRBSI diagnoses.

The registry was approved by the central ethics committee (Magdeburg University Hospital, approval no. 84/14) as well as by local ethics committees of the respective sites.

Registry data entered until March 2021 only on the jugular and subclavian vein CVC ≥ 1 day in situ were used for the present analysis. Underlying diseases included only hematological malignancies, benign hematological diseases, and germ cell tumors (including patients who received autologous stem cell transplantation [SCT] after high-dose chemotherapy). Only CRBSI classified as *definite* (dCRBSI) or *probable* (pCRBSI) were considered with the combination of both being summarized as dpCRBSI.

**Fig. 1:**
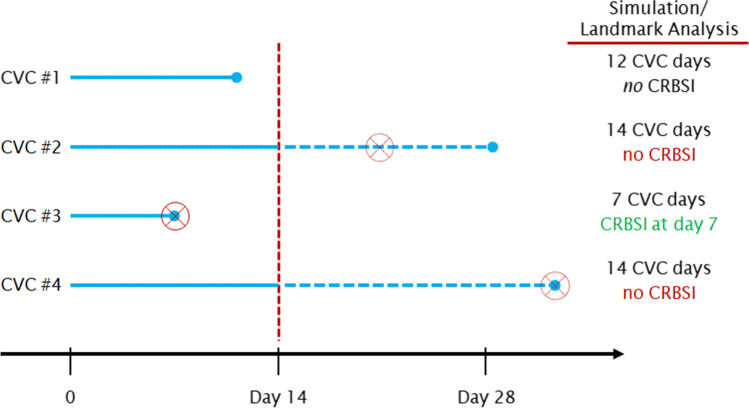
Examples of the procedures and respective simulation analysis carried out for a scheduled CVC removal after 14 days. Dots represent time-points of CVC removal in reality. Crosses with rings represent time-points of CRBSI diagnosis. By day 14, all CVC days and CRBSI events are ignored after this landmark. *CVC* central venous catheter, *CRBSI* CVC-related bloodstream infection

We performed an analysis of simulated CVC removal at 7, 14, and 21 days after CVC insertion. A classic landmark analysis with survival data derived from Kaplan–Meier estimates ignores all events before a time-point [20]. However, our method used here, in a manner of modified and “reverse” landmark analysis, ignores all CVC days and CRBSI events after the landmark of day 7, 14, or 21, respectively, thus simulating CVC removal (see Fig. 1 for examples). Comparable to general epidemiology, incidences were calculated as CRBSI numbers per 1000 CVC days [21], instead of using cumulative incidence estimated with the Kaplan–Meier method. Simulated cases with scheduled CVC removal were compared to real CRBSI cases without simulated premature CVC removal (control group).

### Study endpoints

The primary endpoint was dCRBSI incidence for a scheduled CVC removal after 14 days of CVC insertion (dCRBSI-D14_rmv_). Secondary endpoints were dCRBSI incidence for a scheduled removal after 7 days (dCRBSI-D7_rmv_), and 21 days (dCRBSI-D21_rmv_), as well as dpCRBSI incidence for a scheduled removal after 7, 14, and 21 days (dpCRBSI-D7_rmv_, dpCRBSI-D14_rmv_, and dpCRBSI-D21_rmv_). In addition, the number of CVC needed to be removed (NNR) to prevent one CRBSI was calculated for each endpoint.

### Randomization

Consecutive cases of the registry were stratified per center and randomized in a 1:1:1:1 ratio to either scheduled CVC removal after 7 days (D7_rmv_), 14 days (D14_rmv_), and 21 days (D21_rmv_) or unscheduled CVC removal (control group). For randomization Sealed Envelope™ (London, UK), a web-based platform for randomization and online databases for clinical trials, was used (https://www.sealedenvelope.com/).

### Statistical analysis

In a RCT with neutropenic patients and a median time of CVC in situ of 17 days, the overall dCRBSI rate was 6.0% (37/613) and dCRBSI rate within 14 days after CVC insertion was 3.3% (20/613) [9], showing a rate reduction of about 50%. For the primary endpoint, we therefore assumed a reduction of 50% in the dCRBSI rate for a scheduled CVC removal after 14 days compared to the overall dCRBSI rate. Based on a two-sided type I error α = 0.05, 1492 assessable cases were calculated to reach a power of 1-β = 0.8, meaning 746 cases per group.

For comparison of CRBSI rates, the “N-1” *χ*^2^ test was used as recommended [22, 23]. The CRBSI incidences were compared using the z test.

Statistical analysis was carried out using MedCalc® (Ostend, Belgium), a statistical software package for biomedical research (https://www.medcalc.org/), and IBM® SPSS® Statistics, version 26 (Armonk, NY, USA). Two-sided *p* values < 0.05 were considered statistically significant.

## Results

**Fig. 2:**
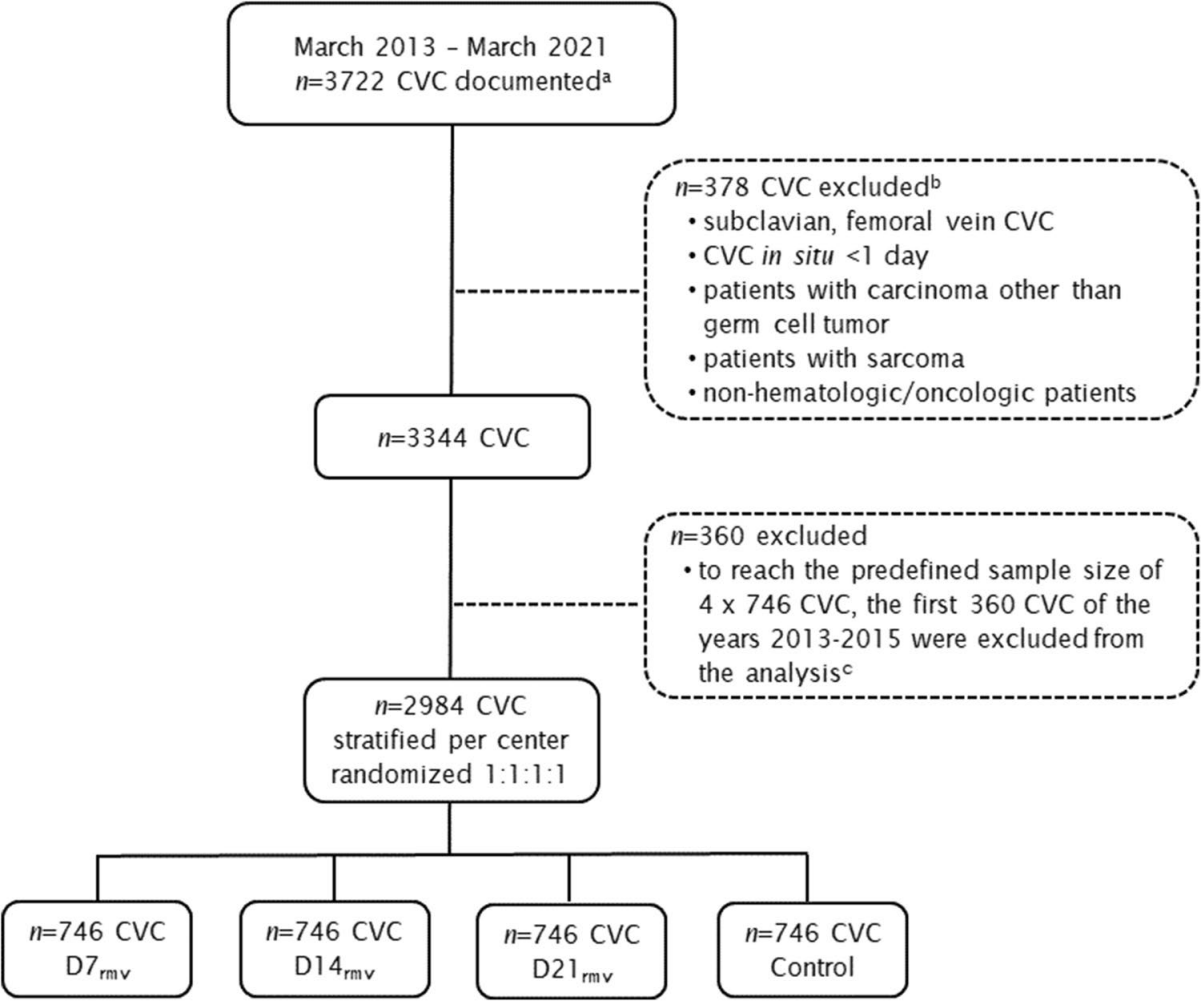
Registry and study enrollment, respectively, and randomization. ^a^non-selected, chronologically according to CVC insertion date, ^b^multiple reasons possible; ^c^the registry consisted of only one site during 2013–2015. *CVC* central venous catheter, *D7*_*rmv*_/*D14*_*rmv*_/D21_rmv_ scheduled CVC removal after 7/14/21 days, *Control* unscheduled CVC removal

A total of 2984 CVC cases were included (Fig. 2). Baseline characteristics and causative pathogens for CRBSI are summarized in Table 1. Patients had a median age of 59 (range 16–95) years and were mostly men (58.8% [1754/2984]). The great majority were patients with hematological malignancies (98.4% [2936/2984]): acute myeloid leukemia in 40.8% (1217/2984), followed by multiple myeloma (21.2% [634/2984]) and non-Hodgkin lymphoma (20.5% [612/2984]). A small fraction of 1.3% (*n* = 39) comprised patients with germ cell tumors, including those who had received autologous SCT after high-dose chemotherapy. Nine (0.3%) patients had a benign hematological disease. Jugular veins were the predominant insertion site (93.2%), about 10% of the CVCs were antimicrobial-coated CVC, and in approximately one-third of the cases, chlorhexidine-coated CVC dressings were used. About every sixth patient (17.6%) was neutropenic at the time of CVC insertion. Baseline characteristics were well balanced between the four groups. In the control group, CVC were in situ for a median of 14 days (interquartile range [IQR] 7–23). In this group, the median time to dCRBSI onset and to dpCRBSI onset was 13 days (IQR 10–20.5) and 12 days (IQR 10–18), respectively. Coagulase-negative staphylococci were the predominant pathogen for dCRBSI and dpCRBSI in all four groups.

**Table 1:** Baseline characteristics

	D7_rmv_ *n* = 746	D14_rmv_ *n* = 746	D21_rmv_ *n* = 746	Control *n* = 746
Patients				
Median age, years (IQR)	59 (49–66)	60 (51–67)	59 (51–66)	59 (48–66)
Men, *n* (%)	425 (57.0)	442 (59.2)	433 (58.0)	454 (60.9)
Underlying disease, *n* (%)				
Acute myeloid leukemia	299 (40.1)	320 (42.9)	288 (38.6)	310 (41.6)
Acute lymphoblastic leukemia	68 (9.1)	58 (7.8)	65 (8.7)	59 (7.9)
Non-Hodgkin lymphoma	146 (19.6)	122 (16.4)	178 (23.9)	166 (22.3)
Multiple myeloma	166 (22.3)	160 (21.4)	166 (22.3)	142 (19.0)
Hodgkin lymphoma	27 (3.6)	28 (3.8)	16 (2.1)	20 (2.7)
Others^a^	40 (5.4)	58 (7.8)	33 (4.4)	49 (6.6)
CVC				
Internal jugular vein, *n* (%)	695 (93.2)	688 (92.2)	697 (93.4)	701 (94.0)
Antimicrobial-coated, *n* (%)	77 (10.3)	69 (9.2)	75 (10.1)	73 (9.8)
CHG-coated dressings, *n* (%)	231 (31.0)	232 (31.1)	235 (31.5)	238 (31.9)
Neutropenia^b^ at insertion, *n* (%)	132 (17.7)	132 (17.7)	124 (16.6)	136 (18.2)
CRBSI				
Causative pathogens, *n*/*N* (%)				
dCRBSI				
CoNS	1/4 (25.0)	14/25 (56.0)	36/42 (85.7)	32/50 (64.0)
* Enterobacteriaceae*	1/4 (25.0)	1/25 (4.0)	1/42 (2.4)	8/50 (16.0)
Other Gram-negative bacteria	0	2/25 (8.0)	2/42 (4.8)	2/50 (4.0)
Other Gram-positive bacteria	1/4 (25.0)	5/25 (20.0)	2/42 (4.8)	6/50 (12.0)
* Candida* spp.	1/4 (25.0)	2/25 (8.0)	1/42 (2.4)	1/50 (2.0)
Multibacterial	0	1/25 (4.0)	0	1/50 (2.0)
dpCRBSI				
CoNS	2/7 (28.6)	29/46 (63.0)	56/68 (82.4)	63/87 (72.4)
* Enterobacteriaceae*	1/7 (14.3)	1/46 (2.2)	3/68 (4.4)	8/87 (9.2)
Other Gram-negative bacteria	0	2/46 (4.3)	2/68 (2.9)	2/87 (2.3)
Other Gram-positive bacteria	3/7 (42.9)	11/46 (23.9)	4/68 (5.9)	9/87 (10.3)
* Candida* spp.	1/7 (14.3)	2/46 (4.3)	2/68 (2.9)	1/87 (1.1)
Multibacterial	0	1/46 (2.2)	1/68 (1.5)	4/87 (4.6)

*D7*_*rmv*_, *D14*_*rmv*_, and *D21*_*rmv*_ cases with scheduled CVC removal after 7, 14, and 21 days, respectively, *IQR* interquartile range, *CVC* central venous catheter, *CHG* chlorhexidine gluconate, *CRBSI* CVC-related bloodstream infection, *dCRBSI* definite CRBSI, *dpCRBSI* combined definite plus probable CRBSI, *CoNS* coagulase-negative staphylococci

^a^Including myeloproliferative neoplasms, myelodysplastic syndromes, aplastic anemias, germ cell tumors

^b^Neutrophils < 500/µL or white blood count < 1000/µL

The dCRBSI rate for scheduled CVC removal after 14 days was 3.5% (25/746) compared to 6.7% (50/746) in the control group (*p* = 0.004). The dCRBSI rate for day 21 removal was 5.6% (42/746; *p* = 0.38), and 0.5% (4/746; *p* < 0.001) for day 7.

Similarly, the dpCRBSI rate for scheduled CVC removal after 14 days was 6.2% (46/746) compared to 11.7% in the control group (87/746; *p* < 0.001). The dpCRBSI rate for day 21 removal was 9.1% (68/746; *p* = 0.10), and 0.9% (7/746; *p* < 0.001) for day 7 removal.

With respect to dCRBSI incidences, no significant differences were identified between scheduled CVC removal after 14 days compared to the control group. dCRBSI-D14_rmv_ was 3.10/1000 CVC days compared to 4.15/1000 CVC days in controls (*p* = 0.23) (Table 2).

**Table 2:** Outcome analyses

	D7_rmv_ *n* = 746	D14_rmv_ *n* = 746	D21_rmv_ *n* = 746	Control *n* = 746
CRBSI incidence, x/1000 CVC days				
dCRBSI	0.86 (*p* < 0.001^a^)	3.10^*^ (*p* = 0.23^a^)	4.10 (*p* = 0.96^a^)	4.15
dpCRBSI	1.50 (*p* < 0.001^a^)	5.71 (*p* = 0.19^a^)	6.64 (*p* = 0.60^a^)	7.22

*D7*_*rmv*_, *D14*_*rmv*_, *D21*_*rmv*_ cases with scheduled CVC removal after 7, 14, and 21 days, respectively, *CVC* central venous catheter, *CRBSI* CVC-related bloodstream infection; *dCRBSI* definite CRBSI, *dpCRBSI* combined definite plus probable CRBSI

^*^Primary endpoint of the study; all other values/calculations are secondary endpoints

^a^*p* values for comparison of the D7_rmv_, D14_rmv_, and D21_rmv_ group, respectively, with the control group

In addition, there was no difference between dCRBSI-D21_rmv_ and the control group, but dCRBSI-D7_rmv_ was significantly lower when compared to the control group (*p* < 0.001). In line with that, dpCRBSI-D7_rmv_ was also significantly lower compared to controls (*p* < 0.001), but no differences could be found for dpCRBSI-D21_rmv_ and dpCRBSI-D14_rmv_ (Table 2).

The calculated NNR for dCRBSI-D14_rmv_, dCRBSI-D21_rmv_, and dCRBSI-D7_rmv_ were 30, 94, and 17, respectively. For dpCRBSI-D14_rmv_, dpCRBSI-D21_rmv_, and dpCRBSI-D7_rmv_, the NNR were 19, 40, and 10, respectively.

By scheduled CVC exchange after 14 days, 52.4% (391/746) of CVC would be removed prematurely by simulation. The respective numbers for day 7 and day 21 are 74.3% (555/746) and 26.4% (197/746).

## Discussion

In this large multicenter, registry-based randomized simulation-study in patients with malignant and benign hematological diseases and germ cell tumors (including those who received autologous SCT) scheduled CVC removal was not superior compared to unscheduled CVC removal regarding definitive CRBSI. As the dCRBSI incidence on day 14 was not lower than in controls, the primary endpoint of the study was not reached.

All NNR calculated for both the primary and secondary endpoints are ≥ 10, not supporting a scheduled CVC removal as only single digit “number needed to treat” values are regarded to be relevant in routine clinical practice, particularly if changes in routine efficacy measures are considered [24]. This indication of unnecessary CVC removal is in accordance with the number of confirmed infections within a large meta-analysis of ICU patients, showing that many CVCs were removed due to suspected infection, but only every ninth case (169/1527) emerged as confirmed infection [25].

Our finding of a reduced dCRBSI incidence by a scheduled CVC removal on day 7 after CVC insertion should not result in changes in clinical practice. CVC in patients with hematological malignancies are generally longer in place given the need for supportive therapies [26] and approximately three-quarters of CVC would be removed unnecessarily.

Unfortunately published studies use different definitions of infectious complications in patients with CVC [27] and different epidemiological key figures like infection rates, incidence, or relative risk, which makes comparisons between epidemiological data difficult. It is therefore recommended to use the CRBSI incidence (incidence density; calculated per 1000 CVC days) rather than the CRBSI rate, to be able to compare CVC length of site use [21].

Thus, dCRBSI-D14_rmv_ was chosen as a primary endpoint for reasons of comparability, as this endpoint was also chosen in a previous RCT on CRBSI in neutropenic patients [9]. Furthermore, a study on 1375 ICU patients, considered CVC use of 15 days or less as short exposure [15], and in a matched-pair analysis comparing data from an RCT with real-world data from our group [28], the median CVC time was 16 days (IQR 9–23). The median time to dCRBSI onset was 14 days (IQR 11–22).

It should be noted that dCRBSI is the most stringent microbiologically based definition of CRBSI [9, 19] yielding consistent epidemiological data [27, 29] that allow for comparison between RCT and real-world studies [28, 29].

Besides procedure-related risks, a major disadvantage of CVC re-insertion in hematological patients is the likelihood of cytopenia at the time of scheduled CVC removal after 7, 14, or 21 days. While neutropenia at the time-point of CVC insertion does not pose a clinically relevant problem [30], thrombocytopenia < 20,000/µL requires prophylactic platelet transfusions [31, 32]. A large number of patients in our cohort had thrombocytopenia < 20,000/µL on day 7, 14, or 21 after CVC insertion (Table 3), indicating the need for prophylactic platelet transfusion – with all the well-known potential risks in case if CVC exchange [32].

**Table 3:** Platelet counts at 7, 14, and 21 days after CVC insertion

	Day 7 *n* = 883	Day 14 *n* = 639	Day 21 *n* = 340
Platelet count, x/µL			
Mean ± standard deviation	92,010 ± 86,019	35,790 ± 64,375	71,900 ± 96,559
Range	1000–533,000	1000–826,000	6000–590,000
Median	72,000	20,000	30,000
Interquartile range	25,000–123,000	12,000–33,000	16,000–89,000
Platelet count < 20,000/µL, *n*/*N* (%)	178/883 (20.2)	315/639 (49.3)	109/340 (32.1)

Data derived from the whole Magdeburg cohort of the SECRECY registry; the same inclusion criteria as stated in the main text. Per every inserted CVC, the platelet count on days 7, 14, and 21 after CVC insertion was documented if the CVC was still in situ on these days

*CVC* central venous catheter

In addition, CVC insertion is associated with a high symptom burden in cancer patients as up to 57% of patients experience pain, pressure and burning, or other generalized symptoms such as fatigue, distress, and drowsiness; symptoms were reported to be severe in almost one-third of patients undergoing CVC insertion [33]. In our cohort, a median of 2 (range 1–12) CVC insertions per patient would be required if a scheduled CVC removal on day14 would have been performed.

While conventional RCT are considered to be one of the most powerful tools clinical researchers possess, we nonetheless used the instrument of a registry-based randomized simulation-study to answer our key question [34].

In conclusion, in patients with hematological diseases and those who received autologous SCT, a scheduled CVC removal after 14 days does not result in a lower CRBSI incidence as compared to unscheduled CVC removal, and can thus not be recommended.

## Data Availability

The data that support the findings of this study are available from the corresponding author upon reasonable request.

## Declarations

### Clinical trial registration

SECRECY (Study to Evaluate Central Venous Catheter-related Infections in Hematology and Oncology) was registered in the German Clinical Trial Register (DRKS; no. DRKS00006551).
